# Privacy-Oriented Technique for COVID-19 Contact Tracing (PROTECT) Using Homomorphic Encryption: Design and Development Study

**DOI:** 10.2196/26371

**Published:** 2021-07-12

**Authors:** Yongdae An, Seungmyung Lee, Seungwoo Jung, Howard Park, Yongsoo Song, Taehoon Ko

**Affiliations:** 1 Desilo Inc Seoul Republic of Korea; 2 Department of Industrial Engineering Seoul National University Seoul Republic of Korea; 3 Department of Computer Science and Engineering Seoul National University Seoul Republic of Korea; 4 Department of Medical Informatics College of Medicine The Catholic University of Korea Seoul Republic of Korea

**Keywords:** COVID-19, homomorphic encryption, privacy-preserving contact tracing, PROTECT protocol, GPS data, mobile application, web service

## Abstract

**Background:**

Various techniques are used to support contact tracing, which has been shown to be highly effective against the COVID-19 pandemic. To apply the technology, either quarantine authorities should provide the location history of patients with COVID-19, or all users should provide their own location history. This inevitably exposes either the patient’s location history or the personal location history of other users. Thus, a privacy issue arises where the public good (via information release) comes in conflict with privacy exposure risks.

**Objective:**

The objective of this study is to develop an effective contact tracing system that does not expose the location information of the patient with COVID-19 to other users of the system, or the location information of the users to the quarantine authorities.

**Methods:**

We propose a new protocol called PRivacy Oriented Technique for Epidemic Contact Tracing (PROTECT) that securely shares location information of patients with users by using the Brakerski/Fan-Vercauteren homomorphic encryption scheme, along with a new, secure proximity computation method.

**Results:**

We developed a mobile app for the end-user and a web service for the quarantine authorities by applying the proposed method, and we verified their effectiveness. The proposed app and web service compute the existence of intersections between the encrypted location history of patients with COVID-19 released by the quarantine authorities and that of the user saved on the user’s local device. We also found that this contact tracing smartphone app can identify whether the user has been in contact with such patients within a reasonable time.

**Conclusions:**

This newly developed method for contact tracing shares location information by using homomorphic encryption, without exposing the location information of patients with COVID-19 and other users. Homomorphic encryption is challenging to apply to practical issues despite its high security value. In this study, however, we have designed a system using the Brakerski/Fan-Vercauteren scheme that is applicable to a reasonable size and developed it to an operable format. The developed app and web service can help contact tracing for not only the COVID-19 pandemic but also other epidemics.

## Introduction

### Background

Since the first case of a previously unidentified coronavirus was reported in Wuhan, China, on December 8, 2019, the COVID-19 pandemic has affected the whole world. According to the World Health Organization (WHO), as of January 25, 2021, the number of COVID-19 cases worldwide was about 97 million, 2.1 million of which were reported to have been fatal [1]. As COVID-19 spreads rapidly, the public is experiencing growing anxiety and concern [2]. COVID-19 has incapacitated the existing medical system with its high communicability and fatality rate, and before vaccines became available, the only available countermeasures were traditional control measures, namely, case isolation, contact tracing and quarantine, physical distancing, disinfection, and practicing personal hygiene [3]; Even with vaccines now available, these countermeasures remain highly relevant and essential.

Therefore, it is imperative to understand the disease propagation and timing in order to take appropriate and timely measures. For example, when 97 patients with COVID-19 were confirmed at a call center in South Korea in March 2020, the Korea Centers for Disease Control and Prevention and the local government formed a joint response team and carried out an epidemiologic investigation using contact tracing [4]. At the time, the team identified and analyzed 1145 people and investigated their surroundings to prevent further disease transmission. Thanks to such active efforts relating to COVID-19 quarantine, despite the early onset of the COVID-19 pandemic, South Korea shows a significantly smaller number of infected patients and lower fatality rate than many other countries.

### Prior Work

Ferretti et al [5] used a renewal equation formulation to develop a mathematical model for determining the speed and volume for effective screening and contact tracing necessary to stop the spread of epidemics and quantify other propagation routes. According to their study, if the self-isolation of an individual who has been in contact with a patient with COVID-19 is delayed by 3 days, no parameter combinations can achieve epidemic control. The study has mathematically proved that the epidemic can be far more effectively controlled when isolation is executed immediately or with a maximum delay of 1 to 2 days. Accordingly, this study explains that if a contact tracing application is used by a sufficient number of people, an epidemic can be controlled by maintaining temporary information about close proximity among individuals and notifying their recent contacts to initiate isolation.

An active measure against the COVID-19 pandemic requires, for example, telehealth screening and management and remote testing, but privacy regulations may pose barriers to such information dissemination. Accordingly, there are claims that privacy regulations should be relaxed for health information exchange in the context of the COVID-19 pandemic [6]. Despite the effectiveness of COVID-19 patient tracing and contact tracing by using digital tools, there are potential privacy leakage risks [7]. As a matter of fact, there have been privacy infringements in the name of public interest in South Korea during the early days of quarantine, when personal information such as gender, age, residence, and place of work was released altogether, leading to unwanted outing incidents [8].

To resolve such issues, applications and technologies are being developed that digitally execute contact tracing while protecting user privacy [9]. In some cases, GPS or Bluetooth information of mobile device users were collected in a centralized manner while attributing temporary identifiers [10,11]. In addition, there are distributed models that store the personal location history on the local mobile device only and compute the distance if a patient comes in close proximity [12]. Both methods, however, are effective only if a majority of users install the app and allow the transmission of one another’s data, which in turn increases privacy risks [13]. There also exist cryptography solutions for privacy protection, such as the technology developed by Apple and Google, which utilizes secure multiparty computation without relying on a trusted server or sends anonymous encrypted or random messages [14]. The study by Gvili [15], however, claims that the said approach by Apple and Google may be vulnerable to several types of security attacks.

### Study Aims

This study aims to propose the PRivacy Oriented Technique for Epidemic Contact Tracing (PROTECT) protocol for digital contact tracing that offers privacy protection by using homomorphic encryption. The proposed system exchanges location data in an encrypted format between the user and the quarantine authorities. By using a novel secure proximity computation technique, the PROTECT protocol makes it possible to identify whether the user has been in contact with any patient with COVID-19 by using only the encrypted location information. This method differs from the privacy protection technologies used in existing contact tracing systems in that it identifies the contacts with encrypted distances, and thus, it can identify whether the user has been in contact with patients with COVID-19 without exposing the user’s location information. It can be said the proposed system uses a privacy-preserving technique of a higher order. In this paper, we first propose a new algorithm for proximity computation and the PROTECT protocol that utilizes this algorithm. Next, we introduce the quarantine app and web service that we have developed to apply the proposed PROTECT protocol to COVID-19 contact tracing and verify that the proposed protocol is practical through experimentation. Finally, we discuss the key results of the study, how it differs from previous studies, and its limitations.

## Methods

### Overview

The key to a privacy-preserving contact tracing system is to protect the location information of not only the patient but also the user, along with the ability to check for proximity. To achieve this, in this study, we used homomorphic encryption and proximity in a discrete grid system to develop a new, secure proximity computation method, and propose a new protocol called PROTECT that applies such a method to deliver data safely among the user, quarantine authorities, and the patient.

### Secure Proximity Computation

The basic method to check for proximity is to compute the distance between two known locations, but this leads to unnecessary location-related privacy issues [16]. Zhong et al [17] proposed three protocols (Louis, Lester, and Pierre) that achieve privacy-secured proximity computation by employing additive homomorphic encryption. The secure proximity computation used in our proposed PROTECT protocol is inspired by the technique used in the Pierre protocol. The Pierre protocol maps the exact location information to the predefined grid areas and substitutes the proximity calculation problem to the calculation of whether the grids are identical or adjacent. It can help determine whether the two locations are in the same grid or in adjacent grids but does not provide information about the two locations. The PROTECT protocol utilizes homomorphic encryption in a novel way such that it does not expose any information other than proximity, yet it is able to perform a high-level computation that can be put into practice immediately.

#### Homomorphic Encryption and Brakerski/Fan-Vercauteren Scheme

Homomorphic encryption is a cryptosystem that supports computation on encrypted data. The result of encrypted computation is also a ciphertext whose decryption returns the same value as if the operation were performed over plain data. Homomorphic encryption has broad applications in cloud environments since it can be used to outsource storage and computation without data leakage.

In the last decade, there have been significant improvements in the efficiency of homomorphic encryption. Lattice-based schemes such as Brakerski-Gentry-Vaikuntanathan (BGV) [18], Brakerski/Fan-Vercauteren (BFV) [19,20], fast fully homomorphic encryption scheme over the torus (TFHE) [21] and Cheon-Kim-Kim-Song (CKKS) [22] currently yield the best performance in practice, but they provide different functionalities. In this work, we focus on the BFV scheme since the proximity of movement of patients with COVID-19 is calculated in the discrete grid system, which will be discussed later. In this system, the proximity is determined by the operation over integral vectors. The BFV scheme is efficient for vectorized operations over the integers, whereas the CKKS and TFHE schemes are more appropriate for approximate and Boolean computations, respectively. We provide a simplified description of BFV as follows.

The BFV scheme consists of five polynomial-time algorithms Setup, Enc, Dec, Add, and Mult. Note that we use symmetric-encryption, which is faster and has better noise growth compared to the public-key variant.

1. Setup (1^λ^): For the security parameter λ, choose a parameter set and sample a secret key *sk*. Parameters include the dimension *n* and the plain-text modulus *p*.
2. Enc (*sk, m*): It takes as the input the secret key *sk* and a plain-text m = (m_1_,...,m_n_) ∈ (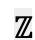
_p_)^n^, which is an *n*-dimensional vector over the finite field 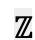
_p_, and returns a ciphertext *c*.
3. Dec (*sk, c*): It decrypts the ciphertext *c* using the secret key *sk* and returns a plaintext *m*.
4. Add (*c, c’*): It outputs the addition of given ciphertexts.
5. Mult (*c, c’*): It performs the multiplication between given ciphertexts and returns the resulting ciphertext.

The BFV scheme satisfies the homomorphic property if parameters are chosen properly. In other words, if *c, c’* are encryptions of *m, m’*, then Add(*c, c’*) and Mult(*c, c’*) are encryptions of *m*+*m’* and *m*⊙*m’*, respectively, where *m*⊙*m’* = (*m_1_m’_1_*,…, *m_n_ m’_n_*) denotes the Hadamard (component-wise) multiplication of two vectors. For simplicity, we will write Add(*c, c’*) = *c*+ *c’* and Mult(*c, c’*)= c * c’.

#### Proximity in Discrete Grid System

In this study, we converted the two location points to a hexagonal grid system and defined that any two points that belong to the same or adjacent grids are “proximate.” The proximity between locations in a continuous space, for example, Euclidean space must be checked with comparison operations; such computation is expensive in a homomorphically encrypted system. The proximity in a discrete space, however, can be computed with a few equality checks, which can be efficiently calculated over encrypted data.

**Figure 1:**
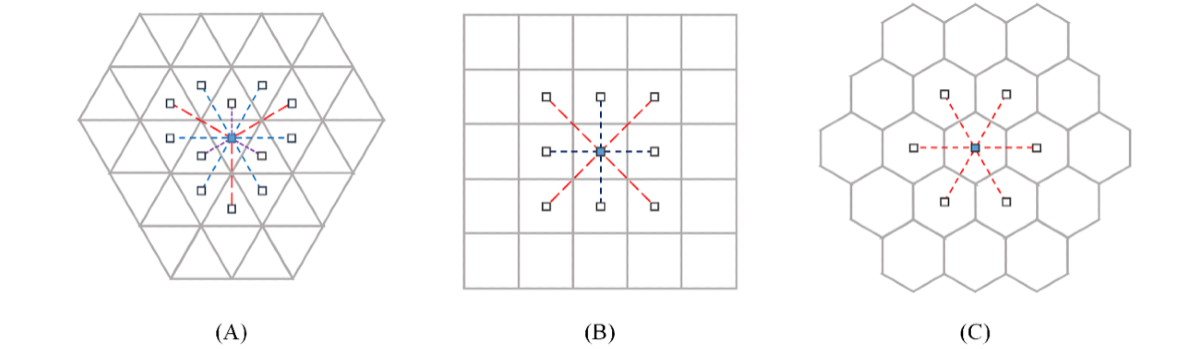
Comparison of (A) triangular, (B) square, and (C) hexagonal grids.

We choose the hexagonal grid system to transform the continuous location information into discrete grids. A hexagonal grid system allows for a simpler definition of neighborhood than triangular or square grids do, so as to reduce the computation overhead. As shown in Figure 1, to define a neighbor, it takes 3 classes in a triangular grid system and 2 classes in a square grid system, but just 1 class in a hexagonal grid system.

**Figure 2:**
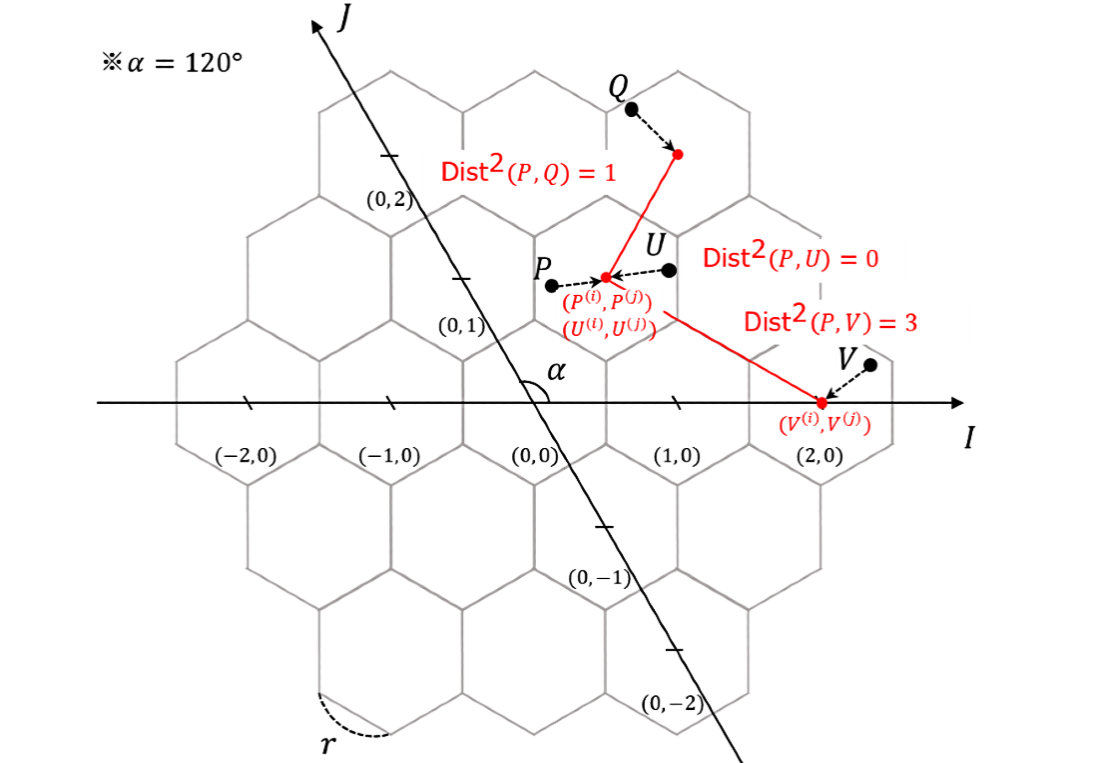
Local *i, j* coordinates of a hexagonal grid system with a side length of *r*.

The transportation network company Uber Technologies Inc introduced a discrete global grid system called Hexagonal Hierarchical Spatial Index (H3) that is based on multiresolution hexagonal grids [23]. As shown in Figure 2, H3 provides the local IJ co-ordinate system for hexagons, which specifies a hexagonal area adjacent to the specified origin with i-axis and j-axis at an angle of 120°.

We denote by *H*: 
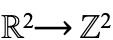
 (*x*, *y*) 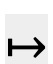 (*i*, *j*) the transformation into the hexagonal grid system with a side length of . In other words, it returns the IJ co-ordinates of the hexagon to which an input point belongs. Some examples are shown in Figure 2.

We also define a metric function as follows:


Dist^2^(∙, ∙): 
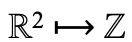
, (P, Q) 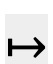 ‖H(P) - H(Q)‖^2^/(3r^2^)


which can be computed as follows:

*Dist*^2^(*P,Q*) = (*P*^(^*^i^*^)^ – *Q*^(^*^i^*^)^)^2^ – (*P*^(^*^i^*^)^ – *Q*^(^*^i^*^)^) (*P*^(^*^j^*^)^ – *Q*^(^*^j^*^)^) + (*P*^(^*^j^*^)^ – *Q*^(^*^j^*^)^)^2^

where *H(P) = (P*^(^*^i^*^)^*, P*^(^*^j)^), H(Q) = (Q*^(^*^i^*^)^*, Q*^(j)^*)* ∈ *Z^2^*.

We use the metric *Dist*^2^ to determine the proximity between two locations. Our definition of proximity in H3 is whether the hexagonal grids corresponding to two locations and are identical or adjacent to each other, or equivalently,

*Dist^2^ (P, Q)* = 0 or *Dist^2^ (P, Q)* = 1.

In the following, we present two properties of *Dist*^2^ to convince that this is a reasonable quantity on which we can make a proper judgement.

**Figure 3:**
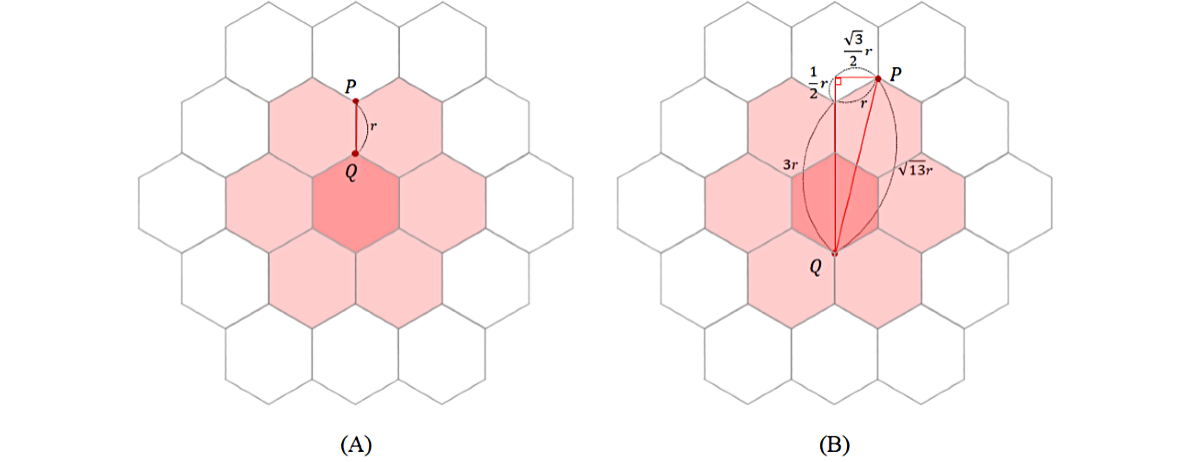
Distance between two points *P* and *Q* on hexagonal grids with a side length of *r*: (A) when the distance between *P* and *Q* is slightly greater than *r* and they are not deemed proximate, (B) when the distance between *P* and *Q* is slightly less than <inline-graphic xlink:href="jmir_v23i7e26371_fig9.png" mimetype="image" xlink:type="simple"/>; and they are deemed proximate.

Figure 3 shows two extreme examples where *Dist^2^ (P, Q)* is relatively large/small compared to the Euclidean distance ‖*P* – *Q*‖. In Figure 3A, the Euclidean distance between two points is but *Dist^2^ (P, Q)* > 1. Meanwhile, we have 
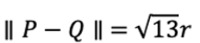
 and *Dist^2^ (P, Q)* =1 in the case of Figure 3B. In application of contact tracing, the primary goal is to detect all contact cases, so the side length should be set sufficiently large based on if-then statements (Textbox 1).

**Relationship between the approximated distance *Dist^2 (P, Q)* and the Euclidean distance ‖P – Q‖**

- If *Dist^2^ (P, Q)*) ≤ 1, then 
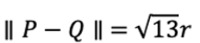
- If ‖P–Q‖ < *r*, then *Dist^2^ (P, Q)*) ≤ 1

In case of a highly contagious epidemic such as COVID-19, a single patient may cause a reproliferation; thus, the examination scope should be rather conservatively set to be broad. The WHO recommends massive testing for all suspected cases of COVID-19 [24]. The Organisation for Economic Co-operation and Development (OECD) also recommends that countries conduct as many tests for COVID-19 as possible, even if they are expensive [25]. The OECD projected that the cost of testing would be much less than the cost of a national lockdown situation [26].

### PROTECT: the Proposed Protocol

#### Protocol Overview

**Figure 4:**
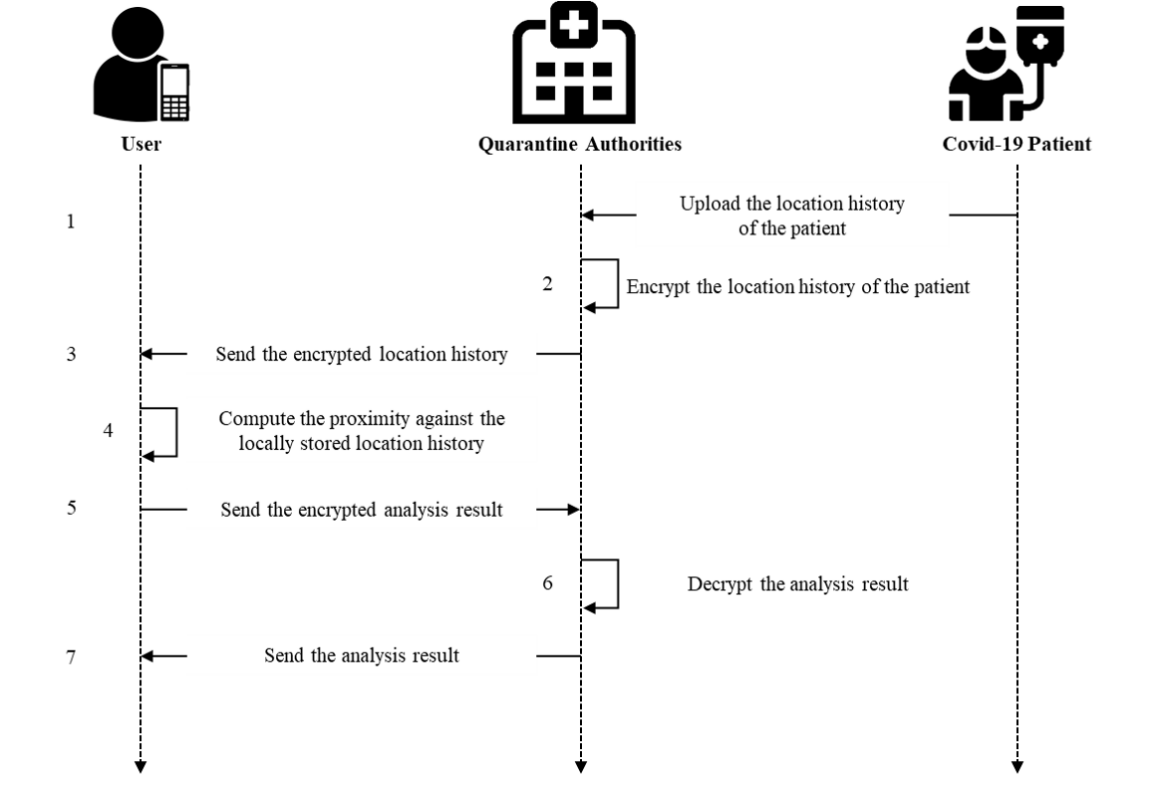
Flowchart of the Privacy Oriented Technique for Epidemic Contact Tracing (PROTECT) protocol.

The proposed protocol PROTECT involves three parties—the user, the quarantine authorities, and the patient with COVID-19. The overall protocol flow is shown in Figure 4. The parties exchange individual ID, timestamps, and GPS locations only.

In this study, we assume that the quarantine authorities are semihonest and that the patient honestly provides their location history to the authorities. The WHO recommends that, as essential surveillance for COVID-19 considering the potential for rapid exponential growth of COVID-19 cases in populations, new cases should be identified, reported, and data included in epidemiological analysis within 24 hours. National authorities should consider including COVID-19 as a mandatory notifiable disease with requirements for immediate reporting [27]. Local governments are already collecting information to track and stop the spread of the coronavirus. The US Centers for Disease Control and Prevention has published a guideline that quarantine personnel shall investigate everyone with whom the patients with COVID-19 have had close contact during the timeframe while they may have been infectious [28], and South Korea collects the location history of patients with COVID-19 and opens them to the public so that those with a high possibility of contact with such patients can voluntarily be examined for COVID-19 [29]. Moreover, it is assumed that all communication in our protocol occurs through a secure channel. When a patient sends personal data to the authorities or the authorities send the encrypted information to the users, such exchange occurs through a secure channel that is invulnerable against a third-party attack (eg, MitM). The definition of each party and further details on the associated events are as follows.

#### Patient With COVID-19

The patient is a user who has been tested positive for COVID-19 and provides 2-week GPS location history to the quarantine authorities. At this time, the location information of the patient is not encrypted.

#### Quarantine Authorities

The quarantine authorities are those individuals who oversee the quarantine system at the municipal or national level. The quarantine authorities receive the location information provided by the patient, encrypt it, and upload it to the server. The encrypted patient location information is then sent to the users who have installed the app. In addition, the quarantine authorities receive the result of the computation at the local user device in the encrypted format, decrypt that result, and then send the decrypted result back to the user. In this process, the quarantine authorities have no access to the personal location information stored on the local user device.

#### User

The user computes, on the individual local device, the proximity between their own location information and the encrypted patient location information received from the quarantine authorities. Here, homomorphic encryption makes it possible to execute computation between the encrypted location information and nonencrypted location information. The computation result is encrypted, as shown in Figure 4. The user then sends the encrypted computation result to the quarantine authorities. The user then receives and checks the decrypted computation result from the quarantine authorities and, in case of high risk of infection, is advised to follow the quarantine protocol recommended by the government.

#### Proximity Computation With BFV and H3

##### Overview

In this section, we provide technical details of proximity computation in the PROTECT protocol. Throughout this section, *P_t_* and *Q_t_* will denote the location data of the patient with COVID-19 and the user, respectively. The location data natively includes time information, but we suppose that data is preprocessed and synchronized so that the elements of the same index have the same timestamps.

Before the protocol starts, the quarantine authorities and the user encode their data locally into the IJ co-ordinates by using the *H*map described in the previous section and generate the vectors as follows:

*P*^(^*^i^*^)^ = (*P*_1_^(^*^i^*^)^, … , *P_n_*^(^*^i^*^)^), *P*^(^*^j^*^)^ = (*P*_1_^(^*^j^*^)^, … , *P_n_*^(^*^j^*^)^) and *Q*^(^*^i^*^)^ = (*Q*_1_^(^*^i^*^)^, … , *Q_n_*^(^*^i^*^)^), *Q*^(^*^j^*^)^ = (*Q*_1_^(^*^j^*^)^, … , *Q_n_*^(^*^j^*^)^), respectively, where *H*(*P_t_*) = (*P_t_*^(^*^i^*^)^, *P_t_*^(^*^j^*^)^) and *H*(*Q_t_*) = (*Q_t_*^(^*^i^*^)^, *Q_t_*^(^*^j^*^)^) for 1≤*t*≤*n*.

##### BFV Encryption

The server sets the parameters for BFV and generates a secret key . The server generates ciphertexts *c*^(^*^i^*^)^←Enc(*sk*, *P*^(^*^i^*^)^) and *c*^(^*^j^*^)^←Enc(*sk*, *P*^(^*^j^*^)^) by using the BFV scheme and sends them to a user.

##### Secure Proximity Computation

On receiving the ciphertexts, the user securely computes the proximity between and . This procedure consists of homomorphic evaluation of the proximity function followed by a ciphertext randomization process.

First, the user homomorphically evaluates *Dist*^2^(*P*, *Q*) by *c_Dist_*^2^ ≔ *d*^(^*^i^*^)^ * *d*^(^*^i^*^)^ – *d*^(^*^i^*^)^ * *d*^(^*^j^*^)^ + *d*^(^*^j^*^)^ * *d*^(^*^j^*^)^ = (*d*^(^*^i^*^)^ – *d*^(^*^j^*^)^)^2^ + *d*^(^*^i^*^)^ * *d*^(^*^j^*^)^ where *d*^(^*^i^*^)^ = *c*^(^*^i^*^)^ – *Q*^(^*^i^*^)^ and *d*^(^*^j^*^)^ = *c*^(^*^j^*^)^ – *Q*^(^*^j^*^)^.

This is an encryption of the vector (*Dist*^2^ (*P_t_* , *Q_t_*))_1≤_*_t_*_≤_*_n_* from the homomorphic property of BFV. Then, the user computes and obtains *c_Prox_* = *c_Dist_*^2^ * (*c_Dist_*^2^ – 1). In our implementation, we performed two homomorphic multiplications after subtraction, added them, and finally performed one relinearization. Note that *c_Prox_* is encrypting *Dist*^2^(*P_t_* , *Q_t_*) * (*Dist*^2^(*P_t_* , *Q_t_*) – 1) = 0 in the th slot, which is zero if and only if *Dist*^2^(*P_t_* , *Q_t_*) = 0 or *Dist*^2^(*P_t_* , *Q_t_*) = 1. Hence, if the user sends *c_Prox_* back to quarantine authorities (the secret key owner), then they would be able to decrypt the ciphertext and determine the proximity of *P_t_* and *Q_t_* by checking if *Dist*^2^(*P_t_* , *Q_t_*) * (*Dist*^2^(*P_t_* , *Q_t_*)–1) = 0 or not. However, this method is not privacy-preserving since the secret key owner can extract more information from the ciphertext *c_Prox_* beyond the proximity.

Hence, the user randomizes the ciphertext *c_Prox_* to solve the issue above. She generates a vector *r* = (*r*_1_, …, *r*_n_) whose entries *r_i_* are sampled independently and uniformly at random from the set 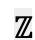
*_p_* *\* {0} *=* {1, 2, …, *p*–1}, and a random encryption of zero *c*_0_ with a large noise parameter. The user outputs the ciphertext *c_RProx_*
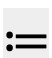
*r* * *c_Prox_* + *c*_0_ and sends it back to the quarantine authorities. Note that the total multiplicative depth of proximity computation is 3.

##### Decryption

The quarantine authorities decrypt *c_RProx_* and obtain *r_t_*’ = *r_t_* * *Dist*^2^(*P_t_* , *Q_t_*) * (*Dist*^2^(*P_t_* , *Q_t_*) – 1) for 1 ≤ *t* ≤ *n*. They conclude that the user has been in contact with a patient at timestamp if this value is zero. We point out that the quarantine authorities learn nothing from the decrypted value about the user data more than the desired result since *r_t_*’ is purely random over 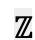
*_p_* \ {0} if *Dist*^2^(*P_t_* , *Q_t_*) ≠ 0, 1. Moreover, the ciphertext *c_RProx_* itself contains no information beyond *r_t_*’ since the user randomized it by *c*_0_. Note that the noise parameter of *c*_0_ should be exponentially larger than that of *r* * *c_Prox_* for security proof.

## Results

In order to apply the PROTECT protocol to COVID-19 contact tracing, we have built a mobile app for patients with COVID-19 and other users, as well as a web service for the quarantine authorities. We also empirically verified the practicality of the PROTECT protocol through performance indicators related to resource consumption such as response time, CPU utilization, and memory consumption on the local device.

### User App

**Figure 5:**
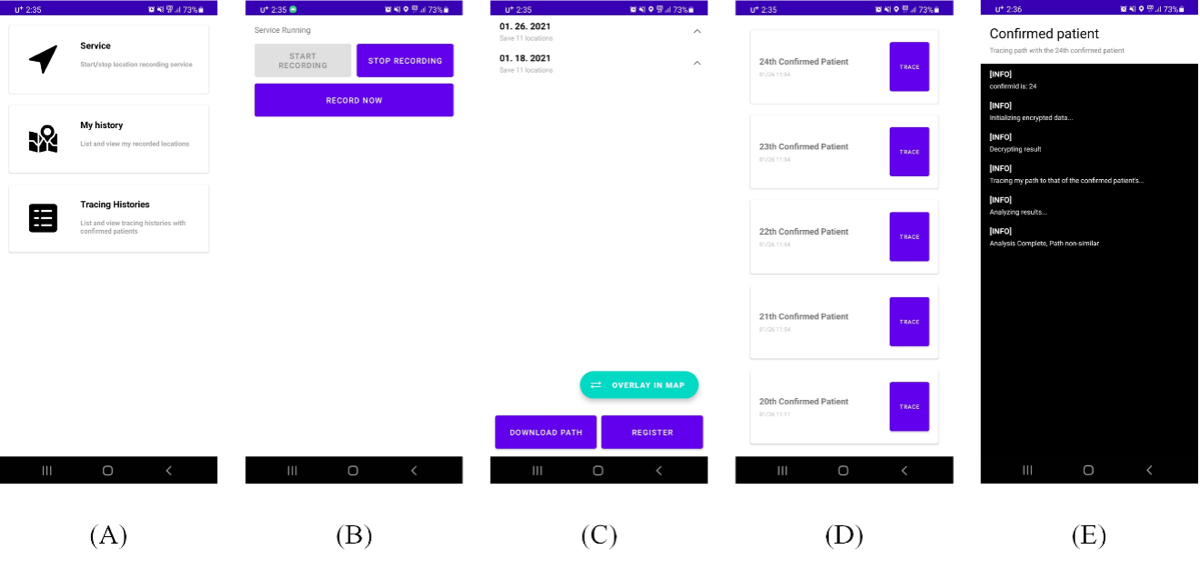
Screenshots of the user application: (A) main screen, (B) GPS data recording setup screen, (C) list of GPS data by day stored on the user's local device, (D) list of encrypted GPS data per patient received from the quarantine authorities, and (E) location history comparison and result.

The smartphone app for the user is as shown in Figure 5. The user can enable or disable the service any time at will (Figure 5B), and easily check the GPS information stored on the local device by date (Figure 5C). Furthermore, the user can view and compare their path with the location information of patients with COVID-19, received as a push message from the quarantine authorities, and check the location details of the potential contact in case the user is suspected to have been in contact with a patient (Figure 5D and 5E).

### Web Service for Quarantine Authorities

**Figure 6:**
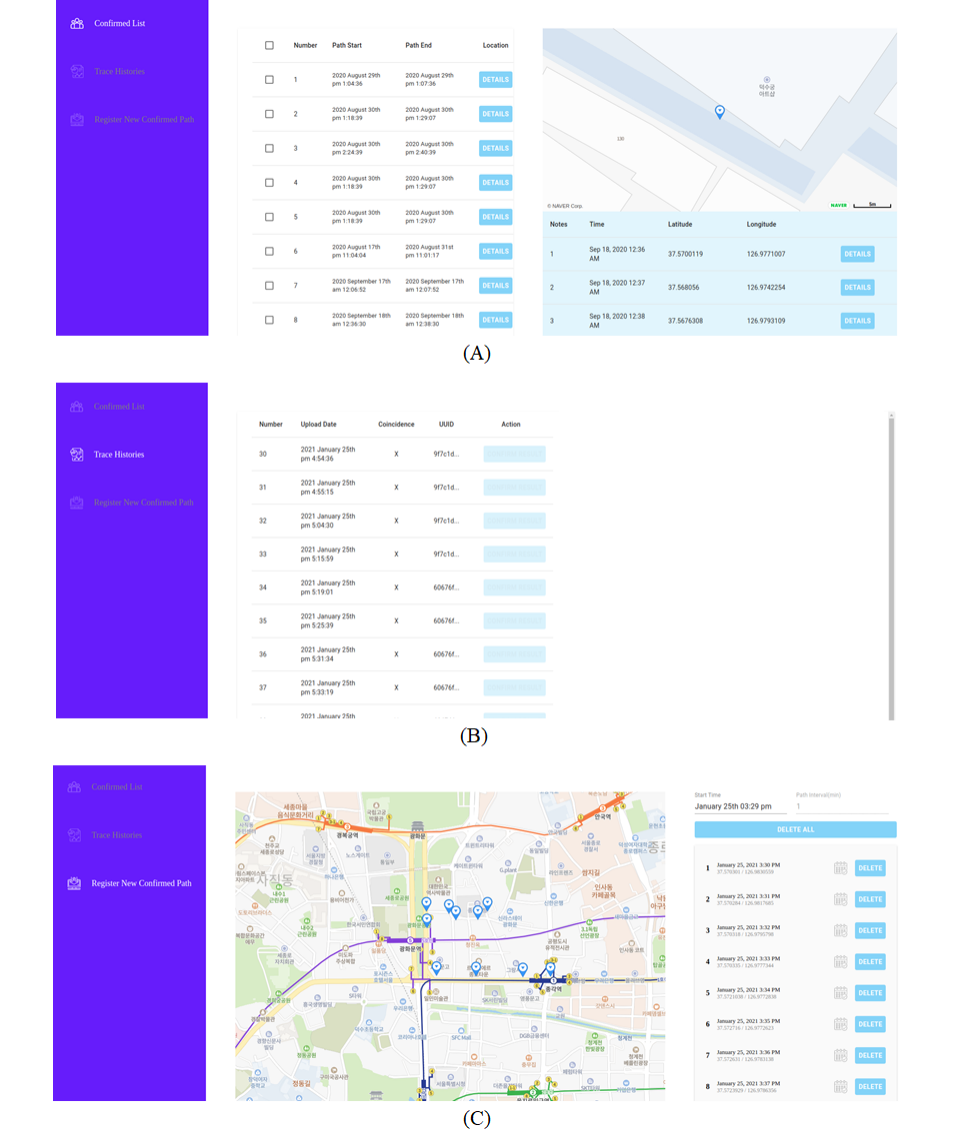
Screenshots of the web service developed for quarantine authorities: (A) list of confirmed patients’ GPS data, (B) list of contact trace histories, and (C) register of new confirmed patients’ GPS data.

The role of the quarantine authorities is to manage COVID-19 patient information and to propagate test results. For this purpose, we built a web service as shown in Figure 6. The quarantine authorities can encrypt the location information provided by the patients and propagate the encrypted information to the users who have installed the app (Figure 6A). Furthermore, although the quarantine authorities have no access to the location information of each individual user, they can analyze the results uploaded by app users and then identify the users whose location history intersects with the location history of registered patients (Figure 6B). We also developed a feature through which the quarantine authorities can easily register patient location history by manually clicking on the map in case the 2-week location data of a patient has not been recorded by the app owing to app nonuse or other reasons (Figure 6C).

### Performance Indicators

To assess the practicality of the app that implements the proposed PROTECT protocol, we installed the developed app on two smartphone models—Samsung Galaxy S20 Plus and Galaxy Note 8—and conducted performance tests. The detailed specifications of the testing devices are as in Table 1.

**Table 1:** Specifications of testing devices.

Specifications	Galaxy S20 plus	Galaxy Note 8
Release Year	2020	2017
Chipset	Samsung Exynos 9 Octa 990	Samsung Exynos 9 Octa 8895
Processor	Octa core (2 × 2.73 GHz Mongoose + 2 × 2.5 GHz Cortex A76 + 4 × 2 GHz Cortex A55)	Octa-core (4 × 2.3 GHz Mongoose M2 + 4 × 1.7 GHz Cortex-A53)
GPU	ARM Mali-G77 MP11	ARM Mali-G71 MP20
RAM	8 GB	6 GB

To satisfy 128-bit security level while maintaining an appropriate size for computation, the base ring dimension was set to 8192, which indicates that the proximity computation for 8192 time points can be executed simultaneously. At the same time, the computation time for the entire data is determined by the size of the base ring dimension. When GPS data is collected every *t* seconds, the number of time points per person collected over the period of 14 days is (60 ⋅ 60 ⋅ 24 ⋅ 14) / *t*. Surely, the larger the value of gets, the smaller the number of time points to be collected per person gets, and the number of comparison computations is also considerably reduced.

It is not necessary to use all time points to compare the time points of the user and the patient. The location information can be trimmed through various methods. It is not necessary to compare all time points for periods where the patient has stayed at a single location for a long time, such as while sleeping or working. In case many patients were present at the same location at the same time, a single computation shall suffice. Furthermore, the occasions wherein the patient has certainly made no contact, such as while driving alone, can also be excluded. Such preprocessing of data can be applied before encryption by the means of epidemiological investigation, when the quarantine authorities collect the location history data of the patients.

If the number of data points refined by the quarantine authorities is *N*, the number of computations (*N_comp_*) is *N_comp_* = ⌈*N*/8192⌉. When the computation time for 8192 time points is *Time_comp_* the total time the proximity computation takes for each user (*Total_Computation_Time*) is *Total_Computation_Time* = *Time_comp_* * *N_comp_*.

As for the proposed PROTECT protocol, the computation times may vary depending on the processing power of the user’s smart device. The test results for computation time in Samsung Galaxy S20 Plus and Note 8 are as presented in Table 2.

**Table 2:** Results of proximity computation tests on testing devices.

Test	Galaxy S20 Plus	Galaxy Note 8
Average CPU utilization (%)	2.158	5.425
Maximum memory consumption during computation (MB)	57.57	58.6
Computation time (*Time*_comp_) (s)	3.246	6.967
Size of encrypted data (MB) (*TransferSize*_Q__→__U_)	1.08	1.08
Size of encrypted data (MB) (*TransferSize*_U__→__Q_)	0.814	0.814

Since S20 Plus has a more powerful processor than that of Note 8, it can be seen that is smaller. When S20 Plus is to compute 1,000,000 encrypted data points received from the quarantine authorities, *N_comp_* =
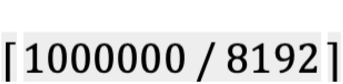
= 123 and *Total_Computation_Time* = *Time_comp_* × *N_comp_* = 3.246 × 123 = 399.258 (seconds). Furthermore, the transfer time between users and quarantine authorities depends on the speed of the network and the size of the transferred data. To account for the difference in the network speed, we checked the size of the transferred data, the results of which are presented in Table 2. In case the quarantine authorities send 8192 encrypted patient location data points to the user, the transferred data size (*TransferSize_Q→U_*) is 1.08 MB on average, and when the user sends the computation result to the quarantine authorities, the data size (*TransferSize_U→Q_*) is 0.814 MB on average. In case of the aforementioned 1,000,000 encrypted data points*, Total_Transfer_Size_Q→U_* = *TransferSize_Q→U_* × *N_comp_* = 1.08 × 123 = 132.84 (MB) and *Total_Transfer_Size_U→Q_* = *TransferSize_U→Q_* × *N_comp_* = 0.814 × 123 = 100.122 (MB).

In addition, the CPU utilization level also varies depending on the processing power of the device. Samsung Galaxy S20 Plus shows a lower average CPU utilization level than that of Note 8. In case of memory consumption during computation, no significant difference was observed. As for CPU utilization and memory consumption, the proximity computation is repeated in batches of 8192, so the increase in the overall time points does not result in several fold increases.

## Discussion

### Principal Results

This study proposed the PROTECT protocol, which utilizes homomorphic encryption to protect privacy while performing digital contact tracing. For this, a novel secure proximity computation technique has been developed so that the location data can be encrypted and exchanged between the user and the quarantine authorities, while the potential COVID-19 patient contact can be identified with encrypted distances only. This method differs from the privacy protection measures used in existing contact tracing systems in that it identifies contacts with encrypted distances, enabling a far higher level of privacy-preserving contact tracing. Our proposed protocol assumes the existence of a centralized organization that already collects the location history of patients and checks for proximity without exposing the location information of the patient to the user or that of the user to the organization. The Bluetooth-based method proposed by Apple and Google requires adoption by a majority of the population for contact tracing to take effect. Our proposed protocol, however, can exhibit the effect of contact tracing for those who have installed the app, no matter how small the number of such users is, provided that the quarantine organization encrypts and provides the patient data collected so far. In addition, the user does not have to provide their location information to the government, which is an advantage against psychological repulsion, one of the greatest hindrances against promoting the use of such an app.

Furthermore, in order to apply the PROTECT protocol to the COVID-19 pandemic, we built a mobile app for patients and users and a web service for the quarantine authorities. In addition, the performance indicators related to resource consumption, such as computation time, CPU utilization, and memory consumption, verify that this protocol is practical enough to be applied to actual COVID-19 quarantine measures.

### Comparison With Contact Tracing in Euclidean Space

Contact tracing in Euclidean space is not secure in terms of privacy. To check for proximity under the Euclidean system, one must first compute the Euclidean distance between the two known locations. This, however, leads to an unnecessary location privacy issue. In order to calculate proximity between the locations of two parties, whoever executes that calculation—be it one of the two parties or an entirely separate third party—must possess the location information of both parties. This implies that at least one party must reveal their location information to another party. On the other hand, as previously discussed, the PROTECT protocol can only determine that two locations are in the same or adjacent grid through secure proximity computation.

**Figure 7:**
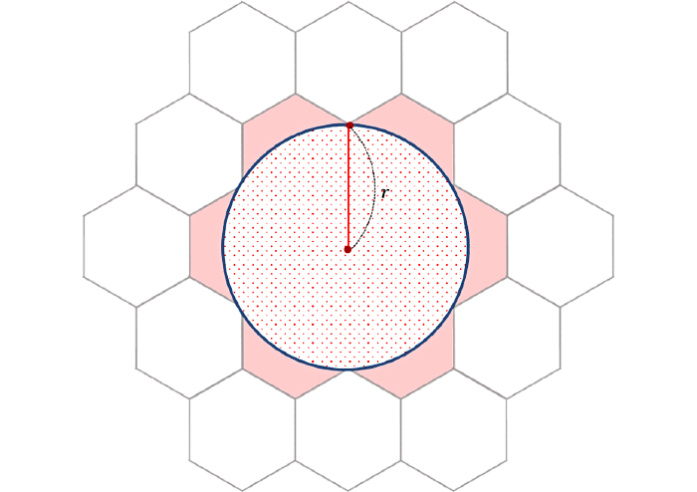
A circle with radius *r* and 7 hexagons with r/2 sides.

Since the hexagonal grid system recognizes a wider range as adjacent than the Euclidean distance method, contact tracing in Euclidean space might appear to be more efficient than the PROTECT protocol. Suppose that we need to test everyone who is within a Euclidean distance of *r* or less from the location of the patient with COVID-19. As shown in Figure 7, if the side length of a single hexagonal grid is *r*/2, the area of 7 hexagonal grids is 
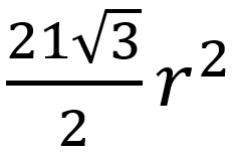
. The area of the circle is π*r^2^*so that the rate (deemed adjacent in the hexagonal grid but not actually adjacent in the Euclidean space) is about 30.9%. If the length of one side of the hexagonal grid is made smaller, this ratio decreases, but the secure computation time in the PROTECT protocol increases. However, the simulation was made under naive but inevitable assumptions.

As COVID-19 is highly contagious, the spread of COVID-19 cannot be covered by the Euclidean space. Rather, the examination scope should be expanded sufficiently. As mentioned earlier, many international organizations are already recommending mass testing for COVID-19 [25-27]. Previous studies have also demonstrated that mass testing is highly effective through COVID-19 epidemic simulation [30,31].

**Figure 9:**
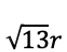
Inline graphic 2.

### Limitations

The proposed protocol and system also have limitations shared by all contact tracing methods that make use of digital technologies. First, there is the limitation of the performance of the smartphone device itself. The accuracy of the GPS location data of each device may vary. GPS, especially, when compared to Bluetooth, is relatively less accurate in an urban setting with many indoor environments and high-rise buildings. Such limitations of the device performance can be complemented by using indoor positioning data such as Wi-Fi and Bluetooth, as well as geomagnetic location measurement techniques. In fact, the indoor positioning data collection technologies have seen much improvement through the advancement of technologies such as fingerprinting.

Second, in this study, all patients with COVID-19 are considered. However, in actual quarantine scenarios, we only need to compare to the patients in the corresponding region, thus reducing the total time taken for the comparison. Third, the homomorphically encrypted computation logic was developed in the same language for both the web server and the mobile app. Thus, there were inefficiencies to make it run within an Android app, such as porting Microsoft SEAL (Simple Encrypted Arithmetic Library) to WebAssembly with a JavaScript interface and then running it on a JavaScript engine within a browser. This should be addressed by directly invoking SEAL C++ APIs (application programming interfaces) using JNI (Java Native Interface) for Android applications. Resolving such inefficiencies would enable the development of a practical solution that is applicable to actual quarantine scenarios.

### Comparison with Prior Work

In order to prevent the location privacy issue related to the calculation of proximity using location information, Gruteser and Grunwald [32] and Bettini et al [33] utilized the concept of k-anonymity for location privacy through dummy data. This method can be a useful means to protect location privacy in various location-based services. However, it is inefficient in the practical setting where the proximity needs to be checked while protecting the location privacy of both parties. In addition, Hu et al [34] proposed a method of calculating the distance using homomorphic encryption and a comparative computation using Geohash. The first method can prevent direct exposure of location information through its use of homomorphic encryption, but location information can be indirectly inferred from the distance information obtained in the end; thus, it cannot be deemed sufficiently safe in terms of location privacy. The second method compares geohash using homomorphic encryption, and it is thus relatively much safer in terms of location privacy. However, it is neither practical nor efficient in terms of computation owing to its use of bitwise homomorphic computation.

In contrast, the secure proximity computation method used in this study substitutes the problem of proximity calculation with the computation of identity or adjacency of two grids by mapping the exact location information to a predefined grid system, and then executes the calculation under homomorphic encryption, thus being safe in terms of location privacy and excellent in terms of computation.

Furthermore, from a system-wise perspective, most existing apps, such as TraceTogether [10] of Singapore or COVIDSafe [12] of Australia, are effective only if the users install the app and allow the exchange of data among one another, and they have the drawback of increased privacy risks. Moreover, the method proposed by Apple and Google is also vulnerable to several types of security attacks [15]. Above all, the previously mentioned methods become effective only when a majority of users use the app. However, with regard to the app and web service based on the proposed PROTECT protocol, even if there is only a single user, that user can effectively identify the occurrences of patient contacts as long as the central quarantine authorities have collected the patient location history.

### Conclusions

The whole world is facing an unprecedented global pandemic situation and is trying to overcome this crisis by all means. Various information technology solutions are being actively suggested in this context. Owing to the potential risk of privacy leaks, however, the adoption rates are low and there has been no case of a *killer app* actively used by many.

In this study, we have described the development of a new proximity computation algorithm that can identify proximity occurrences without exposing the COVID-19 patient location and the user location to one another by homomorphically encrypting the location information. We propose PROTECT, a privacy-preserving contact tracing protocol that uses this algorithm, for use during the current COVID-19 pandemic. In order to apply this protocol to COVID-19 quarantine measures, the proposed protocol has been implemented as a smartphone app for patients and the public and a web service for quarantine authorities. Homomorphic encryption of the BFV scheme is used to design a system applicable to a reasonable scale, and through experiments under various conditions, it has been verified that this service is practical enough to be implemented in a real-world scenario. We hope that this approach that intends to resolve the issue through new technologies contributes to the early discovery and suppression of other potential diseases in future.

## Abbreviations

API: application programming interface
BFV: Brakerski/Fan-Vercauteren
BGV: Brakerski-Gentry-Vaikuntanathan
CKKS: Cheon-Kim-Kim-Song
H3: Hexagonal Hierarchical Spatial Index
JNI: Java Native Interface
OECD: Organisation for Economic Co-operation and Development
PROTECT: Privacy Oriented Technique for Epidemic Contact Tracing
SEAL: Simple Encrypted Arithmetic Library
TFHE: fast fully homomorphic encryption scheme over the torus
WHO: World Health Organization
